# Sigma-1 Receptor Inhibition Reduces Mechanical Allodynia and Modulate Neuroinflammation in Chronic Neuropathic Pain

**DOI:** 10.1007/s12035-023-03717-w

**Published:** 2023-11-03

**Authors:** Simona Denaro, Lorella Pasquinucci, Rita Turnaturi, Cristiana Alberghina, Lucia Longhitano, Sebastiano Giallongo, Giuliana Costanzo, Salvatore Spoto, Margherita Grasso, Agata Zappalà, Giovanni Li Volti, Daniele Tibullo, Nunzio Vicario, Rosalba Parenti, Carmela Parenti

**Affiliations:** 1 Section of Physiology, Department of Biomedical and Biotechnological Sciences, University of Catania, 95123 Catania, Italy; 2 Section of Medicinal Chemistry, Department of Drug and Health Sciences, University of Catania, 95123 Catania, Italy; 3 Section of Biochemistry, Department of Biomedical and Biotechnological Sciences, University of Catania, 95123 Catania, Italy; 4 Section of Pharmacology and Toxicology, Department of Drug and Health Sciences, University of Catania, 95123 Catania, Italy; 5 Unit of Neuropharmacology and Translational Neurosciences, Oasi Research Institute-IRCCS, 94018 Troina, Italy

**Keywords:** Neuropathic pain, Connexin 43, Gap junction, Astrocyte, Microglia, Sigma-1

## Abstract

Neuropathic pain is one of the most debilitating forms of chronic pain, resulting from an injury or disease of the somatosensory nervous system, which induces abnormal painful sensations including allodynia and hyperalgesia. Available treatments are limited by severe side-effects and reduced efficacy in the chronic phase of the disease. Sigma-1 receptor (σ1R) has been identified as a chaperone protein, which modulate opioid receptors activities and the functioning of several ion channels, exerting a role in pain transmission. As such, it represents a druggable target to treat neuropathic pain. This study aims at investigating the therapeutic potential of the novel compound (+)-2*R*/*S*-LP2, a σ1R antagonist, in reducing painful behaviour and modulating the neuroinflammatory environment. We showed that repeated administration of the compound significantly inhibited mechanical allodynia in neuropathic rats, increasing the withdrawal threshold as compared to CCI-vehicle rats. Moreover, we found that (+)-2*R*/*S*-LP2-mediated effects resolve the neuroinflammatory microenvironment by reducing central gliosis and pro-inflammatory cytokines expression levels. This effect was coupled with a significant reduction of connexin 43 (Cx43) expression levels and gap junctions/hemichannels mediated microglia-to-astrocyte communication. These results suggest that inhibition of σ1R significantly attenuates neuropathic pain chronicization, thus representing a viable effective strategy.

## Introduction

Neuropathic pain is a complex and debilitating condition that arises from damage or dysfunction of the nervous system [1]. It is estimated to affect up to 7–10% of the general population and is associated with significant morbidity, impaired quality of life, and increased healthcare costs [2, 3]. The pathophysiology of neuropathic pain is characterized by a combination of peripheral and central sensitization mechanisms leading to abnormal pain processing and perception. Peripheral sensitization involves changes in the excitability of nociceptive neurons in response to tissue damage or inflammation, resulting in increased responsiveness to stimuli that are normally non-painful. Central sensitization, on the other hand, involves changes in the function and plasticity of neurons in the spinal cord and brain that amplify and prolong pain signals, leading to allodynia, pain derived from non-painful stimuli, and hyperalgesia, increased sensitivity to painful stimuli [4, 5].

Emerging evidence suggests that glial cells, including microglia and astrocytes, play a key role in the pathophysiology of neuropathic pain [6, 7]. Indeed, communication between glial cells regulates the homeostasis in the central nervous system (CNS) influencing several functions, including synaptic transmission, inflammatory processes and the development and maintenance of neuropathic pain [8–10].

Gap junctions (GJs) channels, consisting of hemichannels or connexons composed by six connexin (Cx) subunits, represent the biological substrate of direct communication between cells and between cytoplasm and the extracellular environment. GJs enable the rapid diffusion of ions and signal molecules [11]. Cxs are differently and dynamically expressed in CNS cells, influencing cell fate and function. GJs and Cxs appear to be important mediators in the processes of astrogliosis and microgliosis, amplifying and reshaping the reactive responses to stimuli and insults [11]. In particular, Cx43, has been reported to be increased on astrocytes in response to nerve injury and it has been implicated in the development and chronicization of neuropathic pain in the CNS [8, 12, 13]. As such, Cx43 may represents a biomarker and a target candidate in the development of effective therapeutic approaches counteracting neuroinflammation and pain chronicization [14].

The neuroinflammatory processes underlying neuropathic pain contribute to hamper the identification of effective pharmacological therapies. Despite the availability of several treatments for neuropathic pain, there are still significant unmet needs in the field. Current therapies, including opioids, antidepressants and anticonvulsants, are associated with limited efficacy, significant side effects, and a high risk of dependence and abuse [15]. In addition, some patients do not respond to these treatments or experience incomplete relief of their pain symptoms [16]. Therefore, there is a critical need for the development of novel and innovative therapies for the treatment of neuropathic pain.

Sigma receptors (σRs) are a class of non-opioid receptors that are widely distributed throughout the CNS and peripheral nervous system. σRs can be classified into two subtypes of, σ1R and σ2R, which exhibit distinct molecular structure, distribution, and pharmacological profile. σ1R is expressed in several cell types, including neurons and astrocytes, playing a crucial role in multiple cellular functions, such as calcium signalling, lipid metabolism, protein folding and intercellular communication [17]. σ2R is also expressed in the CNS and in peripheral organs, gaining significant attention due to its role in several cellular processes, such as cell growth, differentiation and proliferation [18]. Such a profile has been of particular interest in cancer research, but also in neuroprotection and regeneration, for its role on sphingolipid second messenger in cell proliferation [19, 20].

σ1R is involved in neurological diseases and, in particular, in acute and chronic inflammatory pain disorders [21]. Indeed, studies conducted on animal models of neuropathic pain showed that σ1R antagonists are able to reduce pain hypersensitivity. This suggests that they could represent promising target for drug development [22], potentially capable of overcoming some limitations of current treatments. In this study, we aimed at evaluating the effects of a newly synthesized σ1R antagonist, (+)-2*R*/*S*-LP2, featured by the (+)-*cis*-*N*-normetazocine nucleus [22]. σ1R is able to bind different structural classes of compounds including (+)-*cis*-*N*-substituted *N*-normetazocine derivatives [23, 24], and alazocine ((+)-SKF-10,047), the first compound discovered that highlighted a remarkable σ1R affinity [25]. (+)-2*R*/*S*-LP2, with a *N*-substituent with a 2*R*/*S*-methoxyethyl linker bearing a phenyl ring, displayed nanomolar affinity for σ1R (Ki = 26.61 ± 2.35), with significative selectivity *vs.* σ2R and opioid receptors. In formalin test its antinociceptive effects were prevented by PRE-084 hydrochloride, a known σ1R selective agonist, asserting (+)-2*R*/*S*-LP2 antagonist profile *vs.* σ1R [22].

Here, (+)-2*R*/*S*-LP2, was assayed in the chronic constriction injury (CCI) animal model of neuropathic pain as promising therapeutic agents for the neuropathic pain management. Furthermore, we assessed the efficacy of σ1R antagonism on neurodegenerative process and neuroinflammatory modulation by investigating the reactive state of astrocytes and microglia, and evaluating how the astrocyte-Cx43-microglia axis may be involved in the long-term maintenance of the painful state.

## Materials and Methods

### Animal Model of Neuropathic Pain

All experiments were performed on male Sprague-Dawley rats, purchased from Envigo Laboratories, weighting 200–250 g in accordance with the European Communities Council directive and Italian regulations (EEC Council 2010/63/EU and Italian D.Lgs.no. 26/2014). All efforts were made to replace, reduce and refine the use of laboratory animals. Animals were randomly assigned to different cages (*n* ≤ 3 rats per cage), kept under constant temperature (23 − 25 °C) humidity and light cycle, with ad libitum access to food and water. Rats were randomly divided into three groups: Sham vehicle (*n* = 4), CCI vehicle (*n* = 7) and CCI (+)-2*R*/*S*-LP2 (*n* = 7).

A sciatic nerve chronic constriction injury (CCI) model was established to induce neuropathic pain, according to Bennet and Xie with minor modifications [12, 26]. Briefly, rats were anesthetized with isoflurane inhalation (4% induction, 2% maintenance) and the sciatic nerve was exposed by blunt dissection of the biceps femoris muscle, proximal to the sciatic trifurcation. Four 4/0 chromic silk ligatures were tied around the sciatic nerve until the rat’s hind limb twitched briefly. Sham-operated rats received the same surgical procedures without ligature placement. Finally, the incisions were closed and sutured using 2/0 chromic silk.

### Drug Administration and Pain-Related Behavioural Test

Animals were randomized into three experimental groups: Sham vehicle, CCI vehicle and CCI (+)-2*R*/*S*-LP2. Starting from 9 days post-ligatures (dpl) and up to 16 dpl, rats received a daily intraperitoneal (i.p.) injection of either vehicle or (+)-2*R*/*S*-LP2 (5 mg/kg). Von Frey behavioural test was performed to assess the development of mechanical allodynia at 0 dpl (before surgery) up to 16 dpl. Briefly, rats were located in an elevated plexiglass chamber with a wire mesh bottom and given 20 min to acclimatize, before behavioural testing. The Von Frey filaments, with bending forces ranging from 0.016 to 15 g, were used to vertically stimulate the plantar surface of the ipsilateral hind paw. The “up-down” method was used to determine the withdrawal threshold [27].

### Ex Vivo Tissue Preparation

At 16 dpl, rats were anesthetized using an intraperitoneal injection of ketamine (10 mg/mL) and xylazine (1.17 mg/mL). They were then transcardially perfused with a solution of 0.5 M EDTA (Sigma) in normal saline, followed by ice-cold 4% paraformaldehyde (PFA) in phosphate-buffered saline (PBS). Spinal cords were dissected out and post-fixed with in PFA 4% in PBS at + 4 °C overnight. After PBS washing, tissue samples were cryo-protected with 30% sucrose in PBS at + 4 °C for 3 days. Samples were then embedded in optimum cutting temperature (OCT) medium and rapidly frozen in liquid nitrogen for cryo-sectioning. 20-μm-thick axial sections were collected and mounted on microscopes slide, then stored at – 80 °C until use.

### Immunohistochemistry and Immunofluorescence

Immunofluorescence staining was performed as previously described [28, 29]. Briefly, sections were washed in PBS, then incubated with 10% normal goat serum (NGS, Abcam, Cat.no ab7481, RRID: AB_2716553) or normal donkey serum (NDkS, Abcam, Cat-no ab7475, RRID: AB_2885042) in PBS-0,3% Triton (ThermoFisher Scientific, Cat.no 11488696, CAS: 9002-93-1) for 1 h at room temperature. Then, slides were incubated overnight at + 4 °C with the following primary antibodies, diluted in 1% NGS or 1% NDkS and PBS-0,3% Triton: mouse monoclonal anti-NeuN antibody (Merck Millipore, Cat. No. MAB377, RRID: AB_2298772, 1:100), goat anti-AIF/Iba1 antibody (Novus Biologicals, Cat. No NB100-1028, RRID: AB_521594, 1:100), rabbit polyclonal anti-mouse monoclonal anti-glial fibrillary acidic protein (Gfap) antibody (Santa Cruz Biotechnology, Cat. No. 610566, RRID: AB_397916, 1:100), rabbit polyclonal anti-Cleaved Caspase-3 (Asp175) antibody (Cell Signaling Technology Cat. No. 9661, RRID: AB_2341188, 1:300), rabbit polyclonal anti-Cx43 antibody (Cell Signaling Technology Cat. No 3512, RRID: AB_2294590, 1:100). The following day, samples were washed and incubated for 1 h, at room temperature, with the appropriate fluorescent secondary antibodies, diluted 1:1000 in PBS-0,3% Triton and 1% NGS or NDkS, as follows: goat anti-mouse (Alexa Fluor 488, ThermoFisher Scientific, Cat. No. donkey anti-goat (Alexa Fluor 546, ThermoFisher Scientific, Cat. No. A-11056, RRID: AB_142628), goat polyclonal anti-mouse (Alexa Fluor 488, ThermoFischer Scientific, Cat. No A-11001, RRID: AB_2534069), goat polyclonal anti-mouse (Alexa Fluor 546, ThermoFischer Scientific, Cat. No A-11003, RRID: AB_2534071) goat polyclonal anti-rabbit (Alexa Fluor 488, ThermoFischer Scientific, Cat. No A-11008, RRID: AB_143165), goat polyclonal anti-rabbit (Alexa Fluor 647, ThermoFischer Scientific, Cat. No A-21244, RRID: AB_2535812). Nuclei were counterstained with DAPI 1:1000, diluted in PBS. Slides were then coverslipped with Fluoromount Aqueous Mounting Medium (Sigma-Aldrich, Cat.No F4680). Digital images were acquired using Leica TCS SP8 confocal microscope.

For immunohistochemical analysis, spinal cord sections were washed in PBS, then blocked with 3% H_2_O_2_ in PBS for 15 min at room temperature. Slides were washed three times in PBS, then incubated for 1 h at room temperature with the following primary antibodies: rabbit polyclonal anti-mouse monoclonal anti-glial fibrillary acidic protein (Gfap) antibody (Santa Cruz Biotechnology, Cat. No. 610566, RRID: AB_397916, 1:100) and goat anti-AIF/Iba1 antibody (Novus Biologicals, Cat. No NB100-1028, RRID: AB_521594, 1:100). Afterward, samples were washed in PBS-0,3% Triton, then incubated with biotinylated secondary antibody, diluted in PBS containing 1% bovine serum albumin (Horse Anti-Mouse/Rabbit/Goat IgG Antibody (H + L), Cat.No. BA-1300, RRID: AB_2336188, 1:200). After a 5 min wash, slides were incubated with VECTASTAIN Elite ABC-HRP Reagent (Vector Laboratories, Cat.No PK-7100) for 30 min, at room temperature. Samples were then washed three times in PBS, then exposed to a solution of 1% DAB, 0.3% H_2_O_2_ in PBS until a brown coloration appeared. Nuclei were counterstained with Mayer’s Hematoxylin Solution (Sigma-Aldrich, Cat.No. MHS32). Sections were subsequently dehydrated using a series of increasing ethanol concentrations (50%, 70%, 95%, and 100%) and then cleared with xylene, then coverslipped with Eukitt (Bio Optica, Cat. No. 09–00250). Digital images were acquired using the Nexcope NIB600 biological microscope.

### qRT-PCR

mRNA was extracted incubating spinal cord samples with TRIzol reagent (Invitrogen, Cat. No. 15596026) for 10 min at room temperature. Equal amounts of RNA were reverse transcribed to cDNA using the High-Capacity cDNA Reverse Transcription Kit (ThermoFisher Scientific, Cat. No. 4368814) according to the manufacturer's instructions. Quantitative real-time PCR was then performed using the SYBR Green PCR MasterMix (Life Technologies, Cat. No. 4309155) on the Step-One Fast Real-Time PCR system from Applied Biosystems. The relative mRNA expression level was determined by calculating the threshold cycle (Ct) value of each PCR product and normalizing to a housekeeping gene, using the comparative 2^−ΔΔCt^ method. Rats’ specific primers designed for Il6, Tnf and Gja1 are listed in Table 1.

**Table 1:** Primers sequences used for qRT-PCR analysis

Target	Forward (5’—3’)	Reverse (5’—3’)
*Tnf*	5’-ATGGGCTCCCTCTCATCAGT-3’	GCTTGGTGGTTTGCTACGAC
*Il6*	GCCCACCAGGAACGAAAGTC	TGGCTGGAAGTCTCTTGCGG
*Gja1*	GAAAGAGAGGTGCCCAGAC	GCCAGG TTGTTGAGTGTTAC
*Actb*	ATCCCATCACCATCTTCCAG	ATGAGTGTCCTTCCACGATACCA

### Quantifications and Statistical Considerations

The number of NeuN/Cl Casp3, Gfap/Cl Casp3, and Iba1/Cl Casp3 double-positive cells was quantified by counting the number of double-positive cells per mm^2^ of randomized regions of interest (ROI) from *n* = 4 dorsal horns of lumbar spinal cords obtained from different animals (*n* = 4).

The fluorescence intensity for Gfap, Iba1, and Cx43 analyses was quantified using ImageJ software (Version 2.9.0 for M) by calculating the MFI per area of randomized ROIs deriving from *n* = 4 dorsal horns of lumbar spinal cords of *n* = 4 rats per group. For each population marker (i.e. Gfap and Iba1) a profile plot was calculated and superimposed to the corresponding Cx43 fluorescence profile plot. In order to highlight proximity and/or co-localization between astrocytes-Cx43 and microglia-Cx43, distances of *n* = 10 peaks were calculated.

For the immunohistochemical quantifications, the number of Gfap/Iba1-positive cells was calculated per unit area of each spinal cord lamina of *n* = 4 samples per group.

All statistical tests were performed in GraphPad Prism (Version 9.5.0 for Mac). Data were tested for normality using Shapiro-Wilk test and subsequently assessed for homogeneity of variance. Data that passed both tests were further analysed using One-way analysis of variance (ANOVA) and Holm–Sidak’s multiple post-hoc test for comparison of *n* > 2 groups. Two-way ANOVA, followed by Holm-Sidak’s post-hoc test for multiple comparisons was used where appropriate. Data are presented as mean ± standard deviation (SD) unless otherwise stated. A value of *p* < 0.05 was considered statistically significant and symbols used to indicate statistical differences are described in figure legends.

## Results

### (+)-*2R/S*-LP2 Reduced CCI-Induced Mechanical Allodynia in Rats

**Fig. 1:**
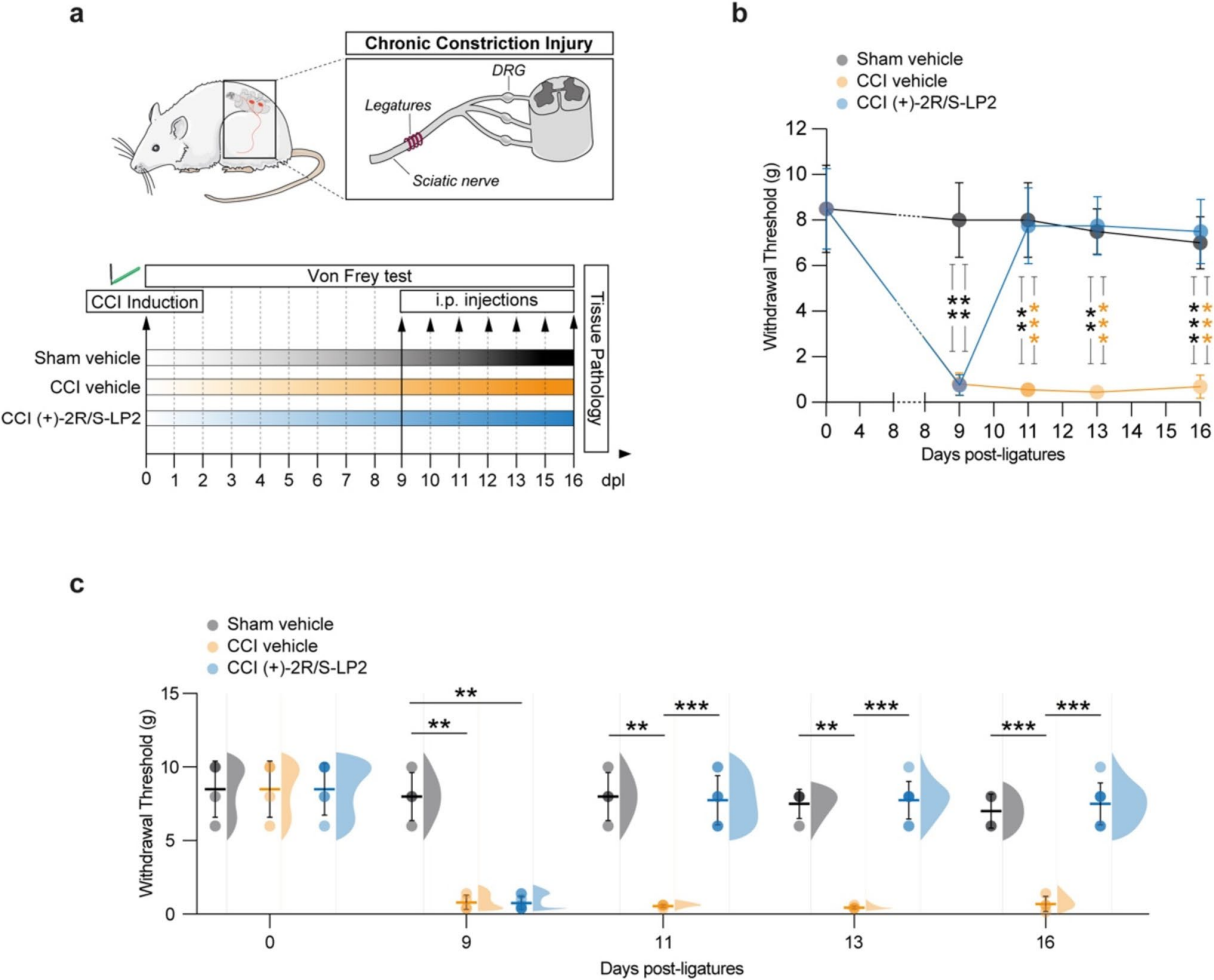
Behavioural analysis of mechanical allodynia in Sham-Vehicle and CCI-treated rats at 0, 9, 11, 13 and 16 dpl. **a** Schematic representation of experimental procedure. **b** Repeated measures of withdrawal threshold following Von Frey filaments stimulation during the time-course of CCI; data are shown as mean (dots) ± SD of *n* = 4 rats per group; black ***p-value < 0.001 and black **p-value < 0.01 versus sham vehicle group; orange ***p-value < 0.001 versus CCI vehicle group; two-way ANOVA and Holm-Sidak’s post-hoc test. **c** Aligned dot plots and frequency distribution curves of Von Frey filaments test in sham vehicle, CCI vehicle and CCI (+)-2*R*/*S*-LP2 rats; data are shown as dots plot ± SD and frequency distribution curves are reported for each group. ***p-value < 0.001, **p-value < 0.01 between groups; two-way ANOVA and Holm-Sidak’s post-hoc test. CCI, chronic constriction injury; dpl, days post-ligatures; SD, standard deviation

To investigate the pathophysiological features of chronic neuropathic pain, we employed the CCI model on male Sprague-Dawley rats, by applying four ligatures to the left sciatic nerve. To assess the onset and the progression of neuropathic pain and to investigate the effects of (+)-2*R*/*S*-LP2 on CCI-induced pain-related behaviour, we performed a time-course analyses by measuring mechanical allodynia, quantified as the withdrawal threshold of the ipsilateral paw to the ligation (Fig. 1a).

Basal mechanical threshold was evaluated before surgery (i.e., 0 dpl) and no significant differences between groups were detected (8.5 ± 2.0 sham vehicle, 8.5 ± 2.0 CCI vehicle and 8.5 ± 1.8 CCI (+)-2*R*/*S*-LP2, Fig. 1b, c).

At 9 dpl, our data indicate the development of mechanical allodynia in CCI-operated rats, as evidenced by a significant reduction of the withdrawal thresholds in both CCI-vehicle and CCI-(+)-2*R*/*S*-LP2 groups, compared to sham-operated animals that maintained high thresholds (0.8 ± 0.5 CCI-vehicle and 0.8 ± 0.5 CCI-(+)-2*R*/*S*-LP2 vs. 8.0 ± 1.6 sham vehicle, Fig. 1b, c). No significant changes in mechanical threshold were observed in sham vehicle rats.

In CCI vehicle animals, the decrease of mechanical threshold persisted up to 16 dpl. On the contrary, daily administration of the σ1R antagonist (+)-2*R*/*S*-LP2 led to a recovery and an improvement of mechanical allodynia, exhibiting an increased withdrawal thresholds as compared to untreated animals at 11 dpl (7.8 ± 1.7 CCI (+)-2*R*/*S*-LP2 vs. 0.6 ± 0.1 CCI vehicle, Fig. 1b, c), 13 dpl (7.8 ± 1.3 CCI L(+)-2*R*/*S*-LP2 vs. 0.5 ± 0.1 CCI vehicle, Fig. 1b, c) and 16 dpl (7.5 ± 1.4 CCI (+)-2*R*/*S*-LP2 vs. 0.7 ± 0.5 CCI vehicle, Fig. 1b, c) comparable to those of the sham-operated group (8.0 ± 1.7, 7.5 ± 1.0 and 7.0 ± 1.2 sham vehicle at 11, 13 and 16 dpl, respectively, Fig. 1b, c).

Overall, our findings indicate that repeated administration of the σ1R antagonist (+)-2*R*/*S*-LP2 effectively alleviates the central sensitization processes that develop during the chronic phase of the disease, leading to the recovery of mechanical allodynia.

### σ1R Targeting Restored CCI-Induced Pro-apoptotic Phenotype on Ipsilateral Dorsal Horn

**Fig. 2:**
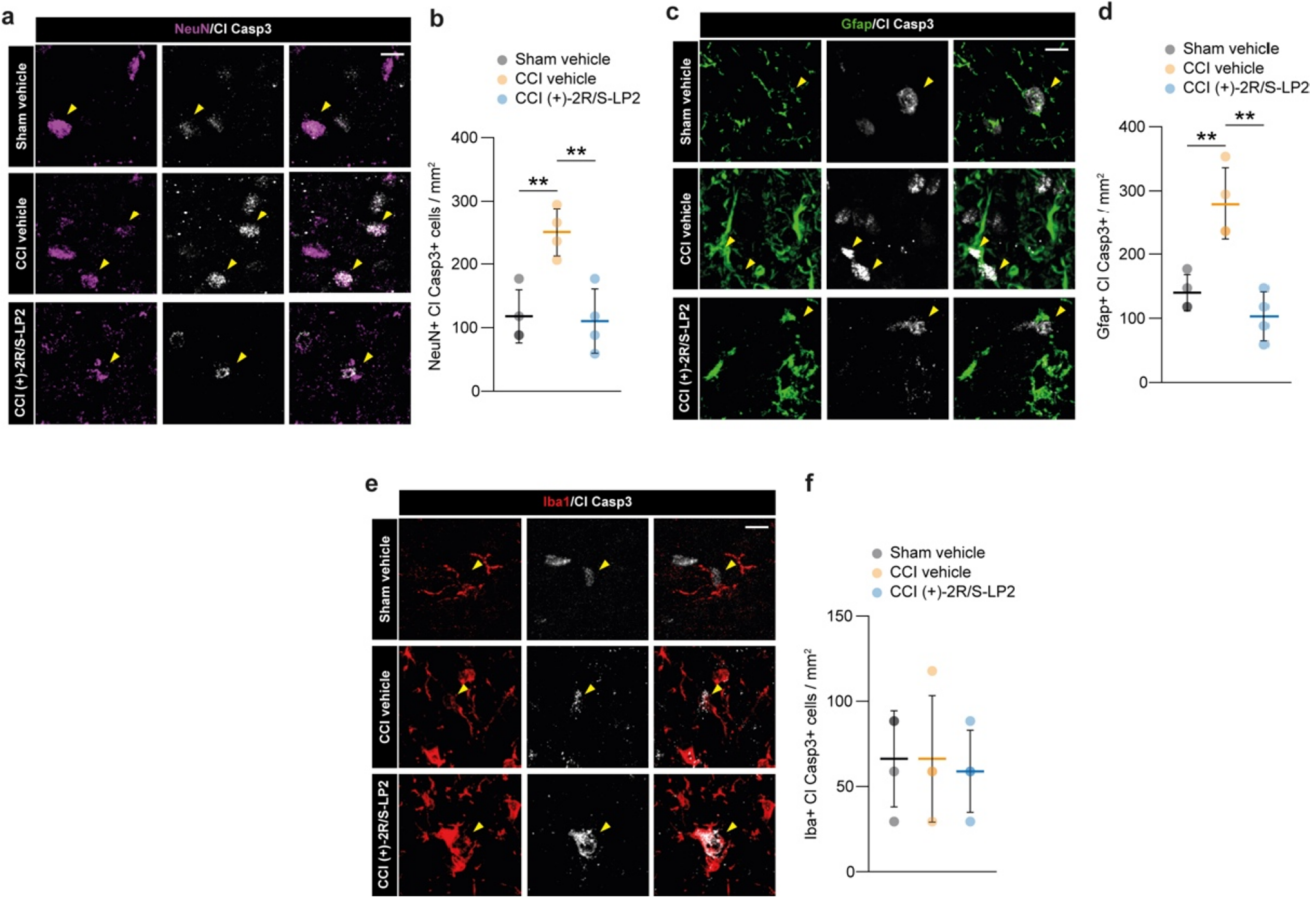
CCI rats show a pro-apoptotic signal on spinal cord resident cell populations. Confocal-assisted representative images. **a**–**b** Representative pictures (**a**) and quantification (**b**) of Cl Casp 3 and NeuN double positive cells per mm^2^; **c**–**d** representative pictures (**c**) and quantification (**d**) of Cl Casp 3 and Gfap double positive cells per mm^2^; **e**–**f** representative pictures (**e**) and quantification (**f**) of Cl Casp 3 and Iba1 double positive cells per mm^2^. Analysis was performed in the ipsi-lateral dorsal horn. Arrowheads in a, c and e indicate Cl Casp3 positive cells. Data are shown as aligned dot plot and mean ± SD of *n* = 4 replicates per group. **p-value < 0.01 between groups; one-way ANOVA and Holm-Sidak’s post-hoc test. CCI: chronic constriction injury; Cl Casp3: cleaved caspase 3; SD: standard deviation. Scale bar: 10 µm

In order to link the (+)-2*R*/*S*-LP2-mediated effects with the beneficial effects at the level of spinal dorsal horns, we performed a cell population analysis on neurons (i.e. NeuN positive cells, Fig. 2a, b), astrocytes (i.e. Gfap positive cells, Fig. 2c, d) and microglia (i.e. Iba1 positive cells, Fig. 2e, f) for cleaved caspase 3 (Cl Casp3) nuclear expression, as a marker of cell suffering and pro-apoptotic signalling. Our data revealed that CCI induced about 2.5 folds increase in NeuN + Cl Casp3 + cells as compared to sham group (250 ± 38 CCI vehicle vs.118 ± 41.7 sham vehicle, Fig. 2a, b), and that (+)-2*R*/*S*-LP2 was able to revert neuronal suffering at 16 dpl (110 ± 50.3 CCI (+)-2*R*/*S*-LP2, Fig. 2a, b). Superimposable results were observed for astroglial cell population, which has been found strongly positive for Cl Casp3 signalling at 16 dpl with an overall increase of Gfap + Cl Casp3 + cells (280 ± 56.4 CCI vehicle vs. 140 ± 28.2 sham vehicle, Fig. 2c, d). Interestingly, (+)-2*R*/*S*-LP2 was able to recover astroglial suffering at 16 dpl in CCI rats as compared to vehicle treated group (103 ± 38.0 CCI (+)-2*R*/*S*-LP2, Fig. 2c, d). Analysis on microglial cells revealed that no significant alteration in the proportion of Iba1 + Cl Casp3 + cells at 16 dpl in ipsilateral dorsal horn (66.3 ± 28.2 sham vehicle vs. 66.3 ± 37.1 CCI vehicle vs. 58.9 ± 24.1 CCI (+)-2*R*/*S*-LP2, Fig. 2e, f).

**Fig. 3:**
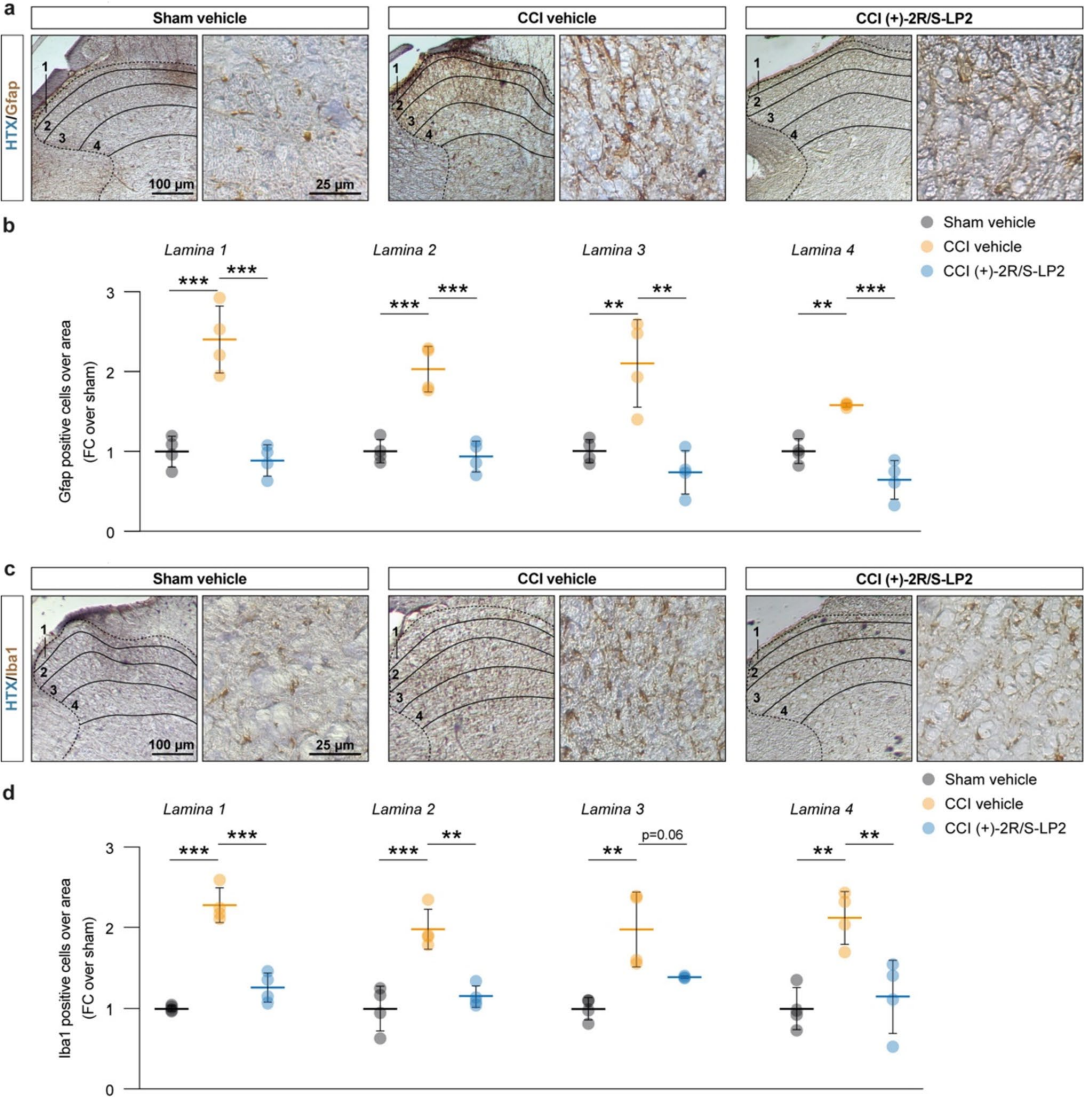
CCI induces a robust astrogliosis in spinal cord dorsal horns, reversed by sigma-1 antagonist (+)-2*R*/*S*-LP2. **a** Representative pictures of Gfap positive cells on ipsi-lateral dorsal horns of the spinal cord indicating laminae 1–4 and representative ROI. **b** Quantification of the number of Gfap positive cells over laminae 1–4 of the lumbar region of the spinal cord. **c** Representative pictures of Iba1 positive cells on ipsi-lateral dorsal horns of the spinal cord indicating laminae 1–4 and representative ROI. **d** Quantification of the number of Iba1 positive cells over laminae 1–4 of the lumbar region of the spinal cord. Data are shown as aligned dot plots and mean FC ± SD of *n* = 4 samples per group. ***p-value < 0.001, **p-value < 0.01 between groups; One-way ANOVA and Holm-Sidak’s post-hoc test. CCI: chronic constriction injury; FC: fold change; HTX: hematoxylin; ROI: region of interest; SD: standard deviation

Given the critical role of glial cells in chronicization of neuropathic pain and their modulatory effects on neuronal fate and response to stressful stimuli, we moved to quantify the proportion of astroglial cells and microglial cells in the ipsilateral laminae. Our immunohistochemical analysis revealed that CCI induces a significant increase of the proportion of astrocytes in lamina 1–4 of both Gfap + , (2.4 ± 0.4 lamina 1 CCI vehicle, 2.0 ± 0.3 lamina 2 CCI vehicle, 2.1 ± 0.6 lamina 3 CCI vehicle, 1.6 ± 0.025 lamina 4 CCI vehicle, FC over sham vehicle, Fig. 3a, b) and Iba1 + cells (2.3 ± 0.2 lamina 1 CCI vehicle, 2.0 ± 0.3 lamina 2 CCI vehicle, 2.0 ± 0.4 lamina 3 CCI vehicle, 2.1 ± 0.3 lamina 4 CCI vehicle, FC over sham vehicle, Fig. 3c, d) as compared to sham operated control. This effect was reverted by (+)-2*R*/*S*-LP2 treatment that restored near-normal levels of glial cell population in ipsilateral laminae 1–4 (0.9 ± 2.0 lamina 1 CCI (+)-2*R*/*S*-LP2, 0.9 ± 0.2 lamina 2 CCI ( +)-2*R*/*S*-LP2, 0.7 ± 0.3 lamina 3 CCI (+)-2*R*/*S*-LP2, 0.6 ± 0.2 lamina 4 CCI (+)-2*R*/*S*-LP2 of Gfap + cells and 1.3 ± 0.2 lamina 1 CCI (+)-2*R*/*S*-LP2, 1.2 ± 0.1 lamina 2 CCI (+)-2*R*/*S*-LP2, 1.4 ± 0.016 lamina 3 CCI (+)-2*R*/*S*-LP2, 1.2 ± 0.5 lamina 4 CCI (+)-2*R*/*S*-LP2, of Iba1 + cells FC over sham vehicle, Fig. 3a–d).

### σ1R Targeting Reverts Il6 and Gja1 Mediated Signalling

**Fig. 4:**
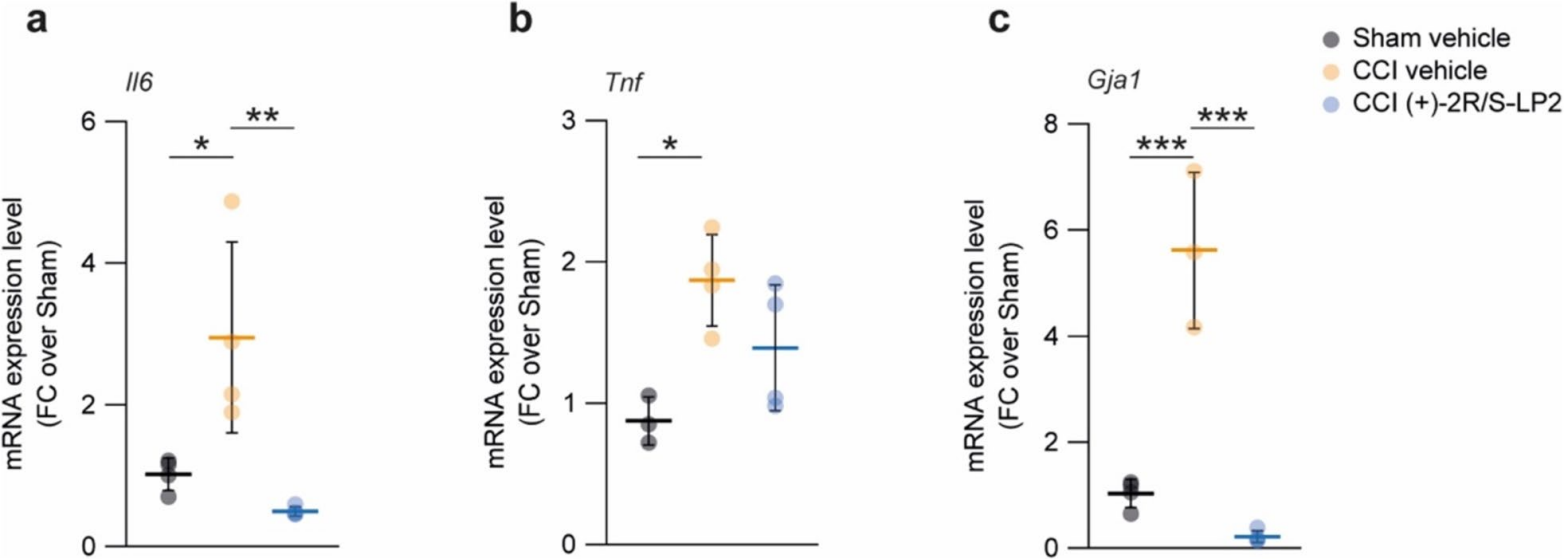
Sigma-1 receptor targeting ameliorates CCI-induced pro-inflammatory milieu. mRNA expression levels of (**a**) Il6, **b** Tnf and (**c**) Gja1 in the spinal cord of Sham vehicle, CCI vehicle and CCI (+)-2*R*/*S*-LP2 rats. Data are shown as dot plots and mean FC ± SD of *n* ≥ 3 samples per group. ***p-value < 0.001, **p-value < 0.01, *p-value < 0.05 between groups; one-way ANOVA and Holm-Sidak’s post-hoc test. CCI: chronic constriction injury; FC: fold change; SD: standard deviation

We then moved to evaluate potential inducers of chronicization of inflammatory stimuli via qRT-PCR analysis on spinal cord biopsies of sham vehicle, CCI vehicle and CCI (+)-2*R*/*S*-LP2 rats. Our data revealed a significant increase of Il6 expression levels in CCI vehicle rats as compared to sham-operated controls (3.0 ± 1.4 CCI vehicle vs. 1.0 ± 0.1 sham vehicle, Fig. 4a), and this effect was abolished by σ1R targeting compound (+)-2*R*/*S*-LP2 (0.5 ± 0.1 CCI (+)-2*R*/*S*-LP2, Fig. 4a). We also evaluated Tnf levels that were found significantly increased of about 2 folds in CCI vehicle and not significantly modulated in treated group (Fig. 4b). Importantly, we observed a strong and significant increase of Gja1, encoding for Cx43, of about 6 folds in CCI rats (5.6 ± 1.5 CCI vehicle vs. 1.0 ± 0.3 sham vehicle, Fig. 4c). Such an increase was reverted by (+)-2*R*/*S*-LP2 treatment at 16 dpl (0.2 ± 0.1 CCI (+)-2*R*/*S*-LP2, Fig. 4c), indicating a potential (+)-2*R*/*S*-LP2-mediated reduction of Cx43-based communication.

### σ1R Targeting Recover CCI-Induced Heterocellular Coupling Mediated by Cx43

**Fig. 5:**
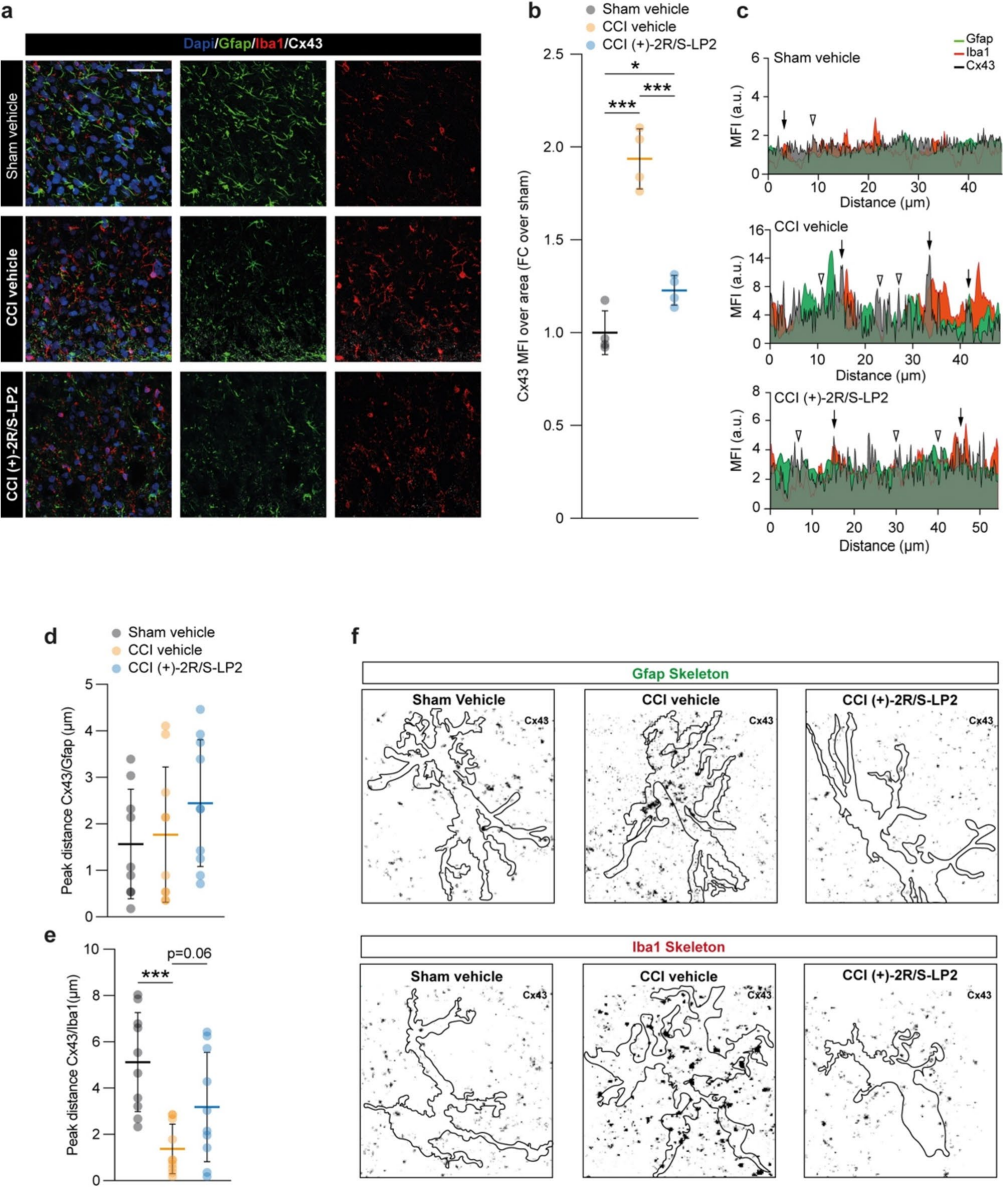
( +)-2*R*/*S*-LP2 disrupts astrocytes-microglia signalling via Cx43 down-regulation. **a** Representative pictures of Gfap, Iba1 and Cx43 immunostaining and (**b**) quantification of the MFI of Cx43 in Sham vehicle, CCI vehicle and CCI (+)-2*R*/*S*-LP2 dorsal horns spinal cords. Data are shown as dot plots and mean FC ± SD of *n* = 4 rats per group (**c**) Profile plot of Gfap, Iba1 and Cx43 MFI expressed as arbitrary unit (a.u.) and (**d**–**e**) peak-distance profile analysis of Cx43/Gfap (**d**) and Cx43/Iba1 (**e**) clusters. Data are shown as dot plots and mean ± SD of *n* ≥ 9 peak-distance per groups. **f** Inverted immunofluorescence pictures displaying Cx43 (black dots) localization analysis on Gfap and Iba1 skeletons, bordered in black. Data are shown as mean ± SD. ***p-value < 0.001, *p-value < 0.05 between groups; One-way ANOVA and Holm-Sidak’s post-hoc test. a.u.: arbitrary unit; CCI: chronic constriction injury; FC: fold change; MFI: mean fluorescence intensity; SD: standard deviation. Scale bar = 25 µm

In an effort to link the observed reduction of Gja1 mRNA levels to the GJ intercellular communication (GJIC) in the spinal cord of CCI rats, we performed an immunofluorescence-based analysis of Gfap positive (i.e. astroglial) and Iba1 positive (i.e. microglia) cells. We stained spinal cord section for Cx43 and we quantified the mean fluorescence intensity (MFI) of Cx43 in the spinal dorsal horns, finding a significant Cx43 MFI increase in CCI rats as compared to sham operated controls (1.9 ± 0.2 CCI vehicle vs. 1.0 ± 0.1 sham vehicle, Fig. 5a, b), and this effect was reverted by (+)-2*R*/*S*-LP2 treatment (1.2 ± 0.1 sham vehicle, Fig. 5a, b). We then moved to assess a peak-distance profile analysis to identify potential Cx43-based homocellular clusters, either between astrocytes or between microglia, or heterocellular clusters, between astrocytes and microglia (Fig. 5c).

Our analysis revealed that in CCI spinal dorsal horns an increased proportion of heterocellular coupling between astrocytes and microglia can be observed as a result of increased colocalization between Cx43 and Gfap or Iba1 peaks (Fig. 5c). Moreover, whether no significant differences in Cx43 and Gfap peak distance between groups (1.6 ± 1.2 sham vehicle, 1.8 ± 1.5 CCI vehicle, 2.5 ± 1.4 CCI (+)-2*R*/*S*-LP2, Fig. 5c, d) were observed, a significant reduced distance between Cx43 peaks and Iba1 peaks in CCI rats was observed as compared to sham operated controls (5.1 ± 2.1 sham vehicle vs. 1.4 ± 1.1 CCI vehicle, Fig. 5c, e), thus indicating a Cx43 involvement in microglia communication. This phenomenon was similarly observed in CCI rats treated with (+)-2*R*/*S*-LP2, but even if a trend can be observed, the reduction was not statistically relevant (3.2 ± 2.4 CCI (+)-2*R*/*S*-LP2, Fig. 5c, e). Our observation was confirmed by analysis of Cx43 localization over Gfap and Iba1 skeleton, which suggested an increased proportion of Cx43 clusters in CCI rats as compared to sham and (+)-2*R*/*S*-LP2-treated rats (Fig. 5f).

**Fig. 6:**
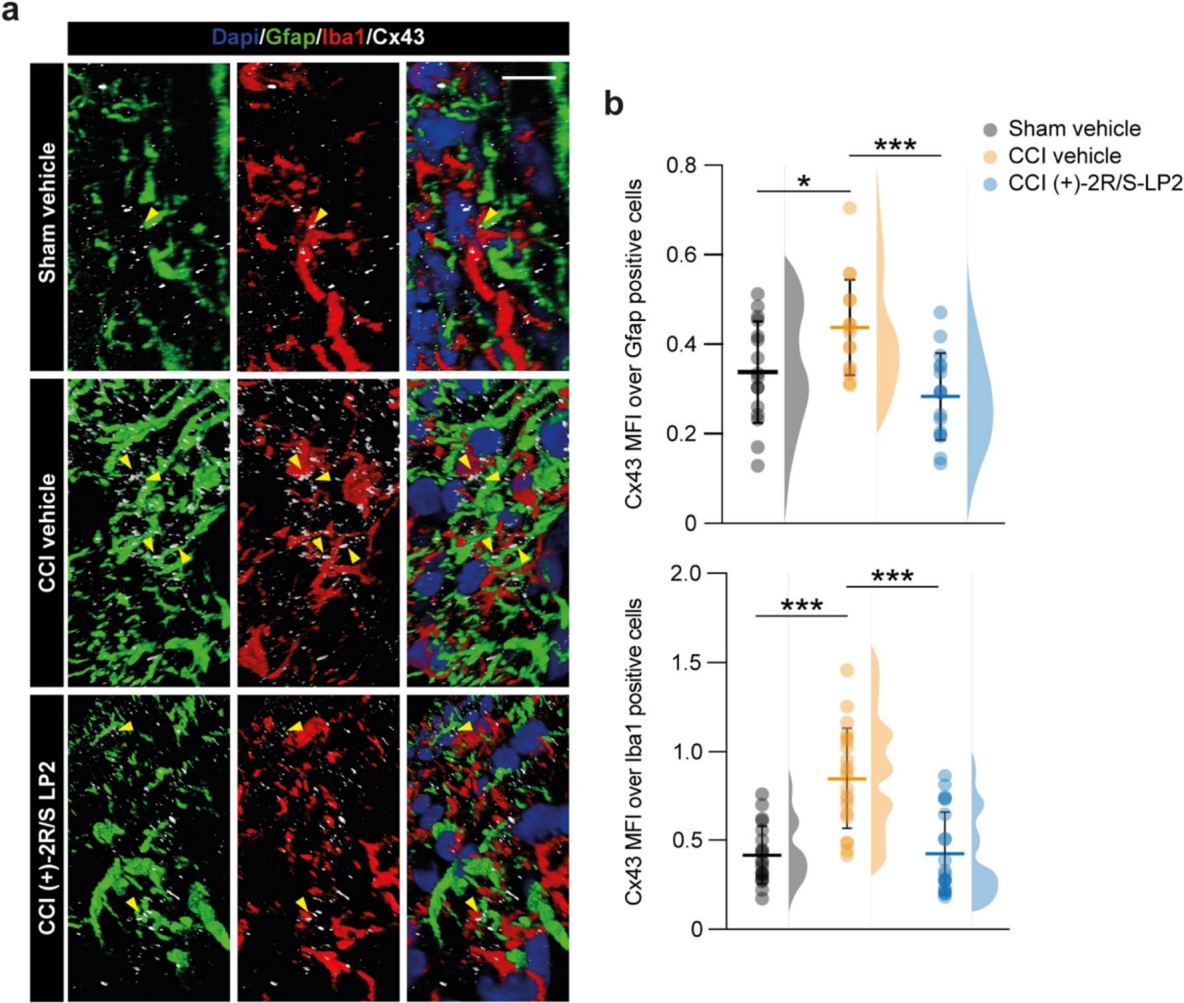
Sigma-1 receptor antagonism modulates heterocellular cell coupling Cx43-mediated. **a** 3D confocal-assisted representative immunofluorescence pictures of spinal cord section of Sham, CCI vehicle and CCI (+)-2*R*/*S*-LP2 treated rats; arrowheads indicate Cx43 positive area between Iba1 and Gfap positive cells. **b** Quantification of Cx43 MFI over area of Gfap- and Iba1-positive cells. Data are shown as dot plot and mean ± SD of *n* ≥ 16 cells per group and frequency distribution curves are reported for each group. ***p-value < 0.001, *p-value < 0.05 between groups; One-way ANOVA and Holm-Sidak’s post-hoc test. CCI: chronic constriction injury; MFI: mean fluorescence intensity; SD: standard deviation. Scale bar = 10 µm

Finally, we quantified Cx43 MFI over Gfap or Iba1 positive area, observing a significant increased proportion of Cx43 in Gfap positive cells (0.3 ± 0.1 sham vehicle vs. 0.4 ± 0.1 CCI vehicle, Fig. 6a, b) and Iba1 positive cells (0.4 ± 0.2 sham vehicle vs. 0.8 ± 0.3 CCI vehicle, Fig. 6a, b) in CCI rats as compared to sham controls. (+)-2*R*/*S*-LP2 treatment was able to revert Cx43 levels in both astrocytes (0.3 ± 0.1 CCI ( +)-2*R*/*S*-LP2, Fig. 6a, b) and microglial cells (0.4 ± 0.2 CCI (+)-2*R*/*S*-LP2, Fig. 6a, b) to near-normal levels.

## Discussion

Neuropathic pain is a chronic condition characterized by a complex and often elusive pathophysiological mechanism. Despite substantial research endeavours, treatments and underlying mechanisms of neuropathic pain continue to present an unsolved challenge.

In this study we evaluated the efficacy of a newly synthesized σ1R antagonist, (+)-2*R*/*S*-LP2, in terms of its analgesic properties. Additionally, we sought to explore its potential in mitigating the complex neuroinflammatory environment, commonly associated with neuropathic pain condition. Our results demonstrate the effectiveness of the σ1R antagonism in reducing mechanical allodynia, a prominent hallmark of neuropathic pain, following CCI. Repeated (+)-2*R*/*S*-LP2 administration induced a pharmacological blockade of the receptor that consistently improves mechanical allodynia in (+)-2*R*/*S*-LP2-treated rats at different time points post-CCI (i.e., 9, 11, 13 and 16 dpl). These results indicate that (+)-2*R*/*S*-LP2 exhibits the potential to modulate the aberrant pain processing pathways associated with the neuropathic pain state, improving sensory hypersensitivity and mechanical allodynia.

Anti-allodynic effects mediated by (+)-2*R*/*S*-LP2 are in line with other studies analysing the role of σ1R in pain processing circuits. σ1Rs are located in key pain modulatory areas, including dorsal root ganglion (DRG) neurons, dorsal spinal cord, thalamus, periaqueductal gray (PAG) and rostroventral medulla (RVM), thus it might play a significant role in remodelling of pain circuits [30]. The absence of neuropathy in CCI mice lacking σ1R gene expression supports the involvement of σ1R in neuropathic pain development [30]. Notably, it has been demonstrated an upregulation of σ1R expression in spinal cord during the induction phase of neuropathic pain, following sciatic nerve constriction [31, 32]. Such an increase could suggest an adaptive response to counteract the enhanced excitability and aberrant signalling associated with neuropathic pain. Specifically, it has been demonstrated that σ1R could influence N-methyl-D-aspartate (NMDA) receptors and Ca^2+^-dependent intracellular cascades involved in pain perception pathways [33, 34]. According to Rodríguez-Muñoz M. et al., this effect may be attributed to the disruption of α_2_δ_1_-NMDA receptor complexes, which are known to play a crucial role in neuropathic pain mechanisms [35–37]. Moreover, Roh D. H. et al., observed that the BD1047 treatment, a well-known σ1R antagonist, blocked the increased NMDA receptor subunit 1 phosphorylation CCI-induced. This post-translational modification enhances receptor sensitivity, prolongs its activation, and augments calcium influx, thereby contributing to the development and maintenance of chronic pain states [31].

Based on our preliminary in vivo results highlighting the (+)-2*R*/*S*-LP2 efficacy in mitigating mechanical allodynia, we delved into elucidating the underlying mechanism responsible for the efficacy of the compound. Particularly, our focus shifted towards its potential impact on glial and neuronal microenvironment in the context of CCI-induced neuropathy. Our study reveals significant alterations of the homeostatic state of glia cells and apoptotic signalling following CCI. Specifically, we observed a robust increase in reactive astrogliosis, as evidenced by elevated number of Gfap- and Iba1-positive cells in the ipsilateral dorsal horn of the spinal cord. Furthermore, immunofluorescence analysis revealed an elevated apoptotic signal characterized by increased double-positive staining for Cl Caspase-3, as a marker of apoptosis, in both astrocytes and neurons of the CCI group as compared to the sham operated group. Interestingly, these pronounced effects of CCI-induced astrogliosis and apoptotic signalling were effectively attenuated by treatment with (+)-2*R*/*S*-LP2. In line with our results, several evidences support the role of σ1R antagonism in modulating astrocyte function and its potential anti-apoptotic effects. Studies have indicated that the σ1R antagonists could inhibit astrocyte activation induced by various insults, such as injury or substances like methamphetamine [38]. The σ1R antagonist BD1047 abrogated the overexpression of both σ1R methamphetamine-mediated and Gfap, associated with astrocyte activation [38]. Moreover, the modulation of astrocytic activity by σ1R antagonists has been linked to the inhibition of nuclear factor-κB (NF-κB) signalling, which contributes to neuroinflammation. Activation of σ1R has been also shown to enhance calcium-dependent intracellular cascades, such as NMDA receptor-mediated calcium influx and IP3-induced calcium mobilization [39, 40]. These mechanisms result in elevated intracellular calcium levels, which represent important contributors to various cellular processes, including dysregulated neuronal survival and apoptosis, likely leading to neurodegenerative diseases [41–43]. Therefore, by blocking σ1R activation, ( +)-2*R*/*S*-LP2 is able to prevent excessive calcium influx and abnormal calcium mobilization, thereby maintaining calcium homeostasis. This protective mechanism may contribute to reduce apoptotic stimuli, preserving cellular integrity and limiting the propagation of neuroinflammation. We did not observe significant variation of Cl Caspase-3 and Iba1 double-positive cells. We concluded that this result may be at least partially related with the relatively low proportion of reactive microglial cells positive for apoptotic markers [44, 45], or with additional mechanism of microglial programmed cell death during chronic neuroinflammation [46].

Activation of astrocytes and microglia is believed to be a defensive response aimed at protecting the nervous tissue and promoting regenerative processes, playing pivotal roles in controlling CNS functions in physiological and pathological contexts [47]. Prolonged or excessive glial activation can lead to amplification of pain signals and maintenance of neuropathic pain [48, 49]. Activated astrocytes and microglia release several inflammatory molecules, such as interleukins, Tnf [50], and reactive oxygen species, which contribute to neuronal sensitization and the development of chronic pain states [51–54]. It is worth noticing that, whether compelling evidence support the role of glial cells in chronic neuroinflammation maintenance, astrocytes have been proposed as responders to pain signals via intimate interaction with surrounding neurons [55, 56]. Moreover, the role of microglia cells in this scenario has been associated with recovery and proposed as a potential therapeutic target for pain management and for neuroinflammatory modulators [57].

In our study, we also observed a significant increase in the number of Gfap- and Iba1-positive cells in the dorsal horns of the spinal cord of CCI rats. Alongside, we observed an increase of Gfap and Iba1 MFI, indicating heightened activation of these cells. By investigating the expression of pro-inflammatory cytokines, we found their upregulation, including Il6 and Tnf, in the spinal cord tissues of CCI-operated animals. Interestingly (+)-2*R*/*S*-LP2 treatment induced a significant reduction in the number of astrocytes and microglia, as well as a decrease in MFI, indicating a dampening of their activation. Furthermore, (+)-2*R*/*S*-LP2 treatment led to a downregulation of pro-inflammatory cytokines, suggesting a modulation of the glial cell-mediated inflammatory response.

Given the critical involvement of glial activation in the neuropathic process, we investigated the involvement of Cx43, typically expressed in astrocytes. Cx43 is responsible for coordinating the glial response and propagating of signalling molecules, particularly during neuroinflammation and neuropathic pain [11, 13]. In our study, we observed a significant upregulation of Cx43 expression in the spinal cord of CCI animals. Such an increase resembled with the activation of astrocytes and microglia, suggesting a potential association between upregulated Cx43 and reactive gliosis observed in neuropathic condition. Furthermore, it has been observed a significant spatial proximity between Cx43-expressing astrocytes and microglia, suggesting their reciprocal crosstalk and response to exchange signalling molecules including pro-inflammatory cytokines, which may contribute to the chronicization of the neuroinflammatory processes. While Cx43-mediated intercellular communication may relate with inflammatory signals propagation, this is just one aspect of the intricate network of interactions that contribute to neuroinflammation [58–61]. Such an event also involves additional ion channels and metabolites, which affecting communication between glial cells in the CNS, and establishing a crucial network controlling the delicate balance between reactive activation, cell suffering and regeneration [11]. This body of evidence highlight the substantial impact of Cxs-mediated channels on the gradual progression and establishment of chronic states within diseases characterized by inflammation and degeneration, shedding light on the potential targets for therapeutic interventions aimed at mitigating the relentless course of these conditions.

Our findings demonstrate that (+)-2*R*/*S*-LP2 treatment leads to a downregulation of Cx43 expression. Such a reduction was associated with the subsequent decrease in reactive astrogliosis, suggesting that (+)-2*R*/*S*-LP2 may modulate the neuroinflammatory response by targeting Cx43-mediated communication between astrocytes and microglia. The mechanism of modulation of Cx43 expression and functioning by the σ1R, could be related to its role as a signalling modulatory chaperone. Indeed, in the CNS, σ1Rs are primarily localized to the membrane of the endoplasmic reticulum (ER) [62]. Upon activation, σ1Rs are able to translocate to the plasma membrane at the ER-plasma membrane junctions, where they engage in protein-protein interactions with different functional proteins, including Cx43, receptors, ion channels, and kinases [30, 63]. Accordingly, by immunostaining and western blot analyses, Choi, S. R. et al. showed the colocalization of Cx43 and σ1R, suggesting a direct relationship between them [64]. This σ1R-Cx43 interaction could interfere with the regulation of Cx43 expression and functioning.

In conclusion, our study provides compelling evidence for the efficacy of (+)-2*R*/*S*-LP2σ1R antagonist, in alleviating mechanical allodynia and modulating neuroinflammation in a neuropathic pain model. The observed reduction in mechanical allodynia suggests that (+)-2*R*/*S*-LP2 has the potential to modulate the aberrant pain processing pathways associated with neuropathic pain. Furthermore, our results indicate that (+)-2*R*/*S*-LP2 treatment effectively attenuated reactive astrogliosis and apoptotic signalling, indicating its potential in mitigating the complex neuroinflammatory milieu commonly associated with neuropathic pain. During the past decade a significant effort has been placed on intrathecal drug delivery systems for refractory chronic pain [65–68]. More in-depth examination of the impacts of (+)-2R/S-LP2 should prioritize its influence on the CNS by exploring additional methods of administration, especially those that provide heightened precision and effects confined to the CNS.

Collectively our findings contribute to supporting the potential of σ1R antagonists as novel therapeutic agents for neuropathic pain management. Future studies are needed to clarify the signalling pathways and molecular mechanisms involved in the modulation of σ1R and Cx43 in neuropathic pain conditions.

## Data Availability

All data generated or analysed during this study are included in this published article.
